# A survey of tandem repeat instabilities and associated gene expression changes in 35 colorectal cancers

**DOI:** 10.1186/s12864-015-1902-9

**Published:** 2015-09-16

**Authors:** Tugce Bilgin Sonay, Malamati Koletou, Andreas Wagner

**Affiliations:** Anthropological Institute and Museum, University of Zurich, Zurich, Switzerland; Institute of Evolutionary Biology and Environmental Sciences, University of Zurich, Zurich, Switzerland; Department of Computer Science, ETH, Zurich, Switzerland; The Swiss Institute of Bioinformatics, Lausanne, Switzerland; The Santa Fe Institute, Santa Fe, NM United States of America

**Keywords:** Tandem repeats, Colorectal cancer, MSI, Microsatellite instability, Expression, Repeat instability, Microsatellite, Cancer pathways, Cancer genes, Wnt signaling pathway, p53 pathway, Methylation, Hypermetyhlation

## Abstract

**Background:**

Colorectal cancer is a major contributor to cancer morbidity and mortality. Tandem repeat instability and its effect on cancer phenotypes remain so far poorly studied on a genome-wide scale.

**Results:**

Here we analyze the genomes of 35 colorectal tumors and their matched normal (healthy) tissues for two types of tandem repeat instability, de-novo repeat gain or loss and repeat copy number variation. Specifically, we study for the first time genome-wide repeat instability in the promoters and exons of 18,439 genes, and examine the association of repeat instability with genome-scale gene expression levels. We find that tumors with a microsatellite instable (MSI) phenotype are enriched in genes with repeat instability, and that tumor genomes have significantly more genes with repeat instability compared to healthy tissues. Genes in tumor genomes with repeat instability in their promoters are significantly less expressed and show slightly higher levels of methylation. Genes in well-studied cancer-associated signaling pathways also contain significantly more unstable repeats in tumor genomes. Genes with such unstable repeats in the tumor-suppressor p53 pathway have lower expression levels, whereas genes with repeat instability in the MAPK and Wnt signaling pathways are expressed at higher levels, consistent with the oncogenic role they play in cancer.

**Conclusions:**

Our results suggest that repeat instability in gene promoters and associated differential gene expression may play an important role in colorectal tumors, which is a first step towards the development of more effective molecular diagnostic approaches centered on repeat instability.

**Electronic supplementary material:**

The online version of this article (doi:10.1186/s12864-015-1902-9) contains supplementary material, which is available to authorized users.

## Background

Microsatellites, short tandem DNA repeats, are among the most variable loci in the human genome. They experience mutations in the copy number of their repeat units at a rate of 10^−3^ to 10^−7^ per cell division [1, 2]. Most such mutations result from replication slippage that escaped the proofreading activity of mismatch repair systems [3]. To date, microsatellite instability – an increased propensity of a microsatellite to suffer length-altering mutations – has been linked to at least 40 monogenic disorders [4, 5]. Such instability is also commonly observed in many cancers, including colorectal, gastric, endometrial, ovarian, and breast cancer [6, 7]. Among them, colorectal cancer, the third most commonly diagnosed cancer in the world, and the second leading cause of cancer-related deaths in western societies [8, 9] shows several phenotypically distinct subtypes. Of these, tumors with microsatellite instable (MSI) phenotype are found in at least 15 % of sporadic colorectal cancers, and almost all hereditary colorectal cancers [10]. MSI tumors differ from other tumors in their gene expression and methylation patterns to a great extent [11–13].

Several studies reported gene expression changes associated with tandem repeat mutations in human carcinomas. For example, a CAG tri-nucleotide repeat associated with prostate cancer has been identified in the first exon of the androgen receptor gene. Expansion of this repeat decreases gene expression, and increases disease incidence and tumor aggression [14]. Another example involves mutations in the promoter of the telomerase reverse transcriptase (TERT) gene, which causes overexpression of the gene, and is a key mechanism behind some types of cancer [15]. In breast cancer, a dinucleotide CA-repeat within the first intron of the epidermal growth factor receptor (*EGFR*) gene correlates with the gene’s transcription levels. Mutant alleles of the highly polymorphic 28 base pair long repeat in the downstream region of the proto-oncogene *HRAS1* significantly increase disease susceptibility for many cancers, including breast cancer, colon cancer, rectal cancer, bladder cancer, and leukemia [16].

Several studies of colorectal adenomas showed that tumors with mutations in different genes have distinctive expression and methylation patterns [13, 17, 18]. The patterns detected from such large-scale gene expression data sets are being used to stratify colorectal tumor subtypes [17, 19]. A study on comparability of gene expression changes in colorectal cancer, based on data produced in various laboratories, showed that on average 95 % of genes show consistent gene expression changes between two major subtypes of colorectal cancer, independent of the data source [20]. Despite many studies on colorectal cancer, current therapeutic approaches cure only a fraction of patients [10, 21], which necessitates a more complete understanding of the kinds of mutations that contribute to tumorigenesis and their impact on tumor phenotypes. Although copy number variations of long DNA stretches, and single nucleotide polymorphisms have received much attention in colorectal tumors [13, 22], a genome-wide analysis of tandem repeat instabilities is currently not available. Most work on repeat instability in colorectal cancer focuses on variation between tumor and matched normal genomes in merely five marker repeats [23], a tiny fraction of the more than 3 million human microsatellite loci [24, 25]. Recent advances in next generation sequencing and accurate repeat genotyping algorithms enabled us to investigate repeat variation in tumor genomes more comprehensively, and to study their potential consequences on gene expression.

Here we analyze tandem repeat variation in 35 colorectal tumors and their matched normal genomes in proximal (near-gene) promoter regions and exons of 18,439 genes, as well as in a smaller subset of genes in known cancer-associated pathways. We find that MSI tumors are significantly enriched for *de novo* repeat gain, repeat loss, and copy number variation in their exonic and promoter regions. Also, tumors, in general are enriched in genes with such repeat instabilities compared to normal tissues. We observe that genes with repeat instability in their promoters tend to be expressed at lower levels. The promoters of genes in most well-studied cancer pathways, including the p53 and Wnt signaling pathways, are significantly enriched in unstable repeats, and those pathway genes with unstable repeats show gene expression alterations consistent with their role in carcinogenesis, whether oncogenic or tumor-suppressive.

## Results

### Abundant repeat gains and losses in tumors compared to normal genomes

We identified genes with tandem repeats in the exons and promoters of 18,439 genes in 35 colorectal tumors and their matched normal genomes (see Methods and Additional file 1: Table S1 and S2). We found that a tumor genome has on average 1510 exon sequences and 4192 promoters with tandem repeats. A normal genome has on average 1475 exons and 4165 promoters with tandem repeats.

**Fig. 1:**
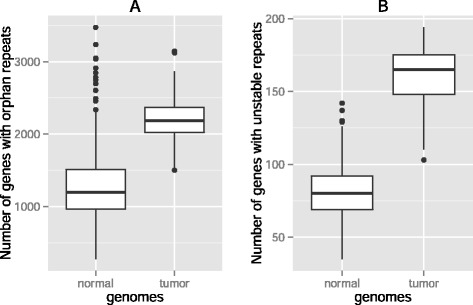
Significantly more promoters with orphan and unstable repeats in tumors. Box plots of the number of gene promoters **a**) with orphan repeats **b**) with unstable repeats in normal-normal genome pairs (left boxes, *n* = 595) and in tumor-matched normal genome pairs (right boxes, *n* = 35) in promoter sequences. Thick horizontal lines in each box mark the median, edges of boxes correspond to the 25th and 75th percentiles, and whiskers cover 99.3 % of the data’s range. The repeat incidences in each pair of boxes are significantly different from each other within each panel (WRS Test, *P* < 10^−16^, *P* < 10^−24^, in panels **a** and **b**)

A mean number of 1043 (one standard deviation: ±337) genes in a tumor genome show repeats that do not occur in the same gene’s promoter in the matched normal genome, compared to a mean number of 1016 (±334) promoter repeats that are specific to normal genomes and do not occur in tumor genomes. In total, there are 2059 (±373) genes that either lost a repeat or gained a *de novo* promoter repeat in a tumor compared to their matched genes in normal genomes. For brevity, we refer to these repeats as *orphans*. Based on several indications of an MSI phenotype reported in [13], we classified four tumors in our data set as MSI (see Methods and Additional file 1: Table S2). These tumors showed a slightly but non-significantly higher number of genes with orphan repeats (2072 ± 376; *P* not significant; Wilcoxon Rank Sum (WRS) test; [26]). We then compared the number of promoters with orphan repeats in the tumor-normal pairs to that of normal genome pairs, based on all possible 595 paired combinations of available normal genomes, and found that significantly fewer genes in healthy tissues contain orphan repeats (1274 ± 481, *P* < 10^−16^, Fig. 1a).

**Fig. 2:**
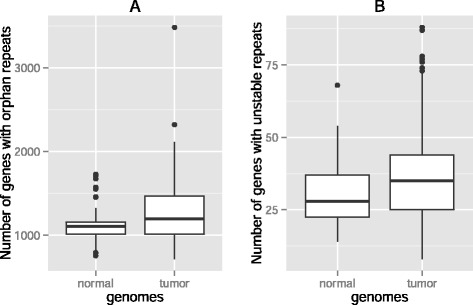
Significantly more genes with orphan and unstable repeats in tumors. Box plots of the number of genes **a**) with orphan repeats **b**) with unstable repeats in normal-normal genome pairs (left boxes, *n* = 595) and in tumor-matched normal genome pairs (right boxes, *n* = 35) in exons. Thick horizontal lines in each box mark the median, edges of boxes correspond to the 25th and 75th percentiles, and whiskers cover 99.3 % of the data’s range. The repeat incidences in each pair of boxes are significantly different from each other within each panel (WRS Test, *P* = 0.01, *P* = 0.04, in panels **a** and **b**)

A similar analysis on exonic repeats revealed that there are, on average, 759 (±372) repeats in a tumor genome that do not occur in the same gene in the matched normal genome, compared to a mean number of 725 (±290) exonic repeats that are specific to the normal genomes and do not occur in the tumor genomes. In total, we found 1320 (±562) genes with orphan repeats in tumor-normal genome pairs, and their number was much higher in MSI tumors (2346 ± 841, *P* = 10^−4^). The number of genes with orphan repeats in tumor-normal pairs was significantly smaller than in normal tissues (1090 ± 147, *P* = 0.01, Fig. 2a). Hence, as in promoters, repeats in exonic regions experience gain and loss incidences more frequently in tumors than in healthy tissues.

### More genes with unstable repeats in tumors

For those genes where both the tumor and matched normal genomes contain a repeat, we next asked how many repeats are *unstable*, that is, varying in the copy number of their repeat unit. Averaged over all 35 tumor-normal genome pairs, the number of genes with unstable repeats is 158 ± 24 (for MSI tumors only: 160 ± 22). This number is significantly greater than the number of genes with unstable repeats in normal genome pairs (81 ± 19, WRS test, *P* < 10^−24^, see Fig. 1b). When we repeated this analysis for exonic repeats, we observed a similar enrichment in repeats that varied in their copy number between the two sets of genome pairs. Specifically, we found on average 36 ± 14 (for MSI tumors only: 40 ± 13, *P* not significant) genes with unstable repeats in a tumor-normal genome pair, a value greater than that in a normal/normal pair (31 ± 12 genes, *P* = 0.04, see Fig. 2b). We conclude that tumor genomes harbor more repeat copy number variation than normal genomes both in their promoters and exons.

### MSI genomes have more genes with repeat instability

**Fig. 3:**
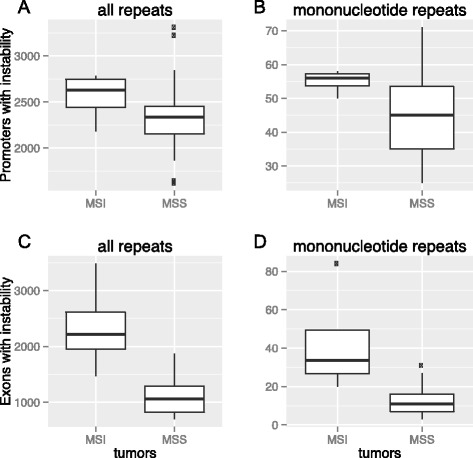
MSI tumors have more genes with repeat instability. Box plots of the number of promoters with instability of **a**) any repeat, **b**) mononucleotide repeats and the number of exons with instability of **c**) any repeat, **d**) mononucleotide repeats in MSI tumors (left boxes, *n* = 4) and in microsatellite stable genome pairs (right boxes, *n* = 31). Thick horizontal lines in each box mark the median, edges of boxes correspond to the 25th and 75th percentiles, and whiskers cover 99.3 % of the data’s range. The repeat incidences in each pair of boxes are significantly different from each other within each panel except for the panel **a** (WRS Test, *P* = 0.029, *P* = 0.003, *P* = 10^−4^, in panels **b**, **c** and **d**, respectively, after Bonferroni correction)

In the previous analyses, we observed an enrichment in the number of genes that contain unstable or orphan repeats in MSI tumors relative to microsatellite stable (MSS) tumors. The differences were, however mostly not significant due to the low number of MSI tumors. To study in greater detail if the MSI phenotype of a tumor genome can help explain differences in repeat instability to its matched normal genome, we decided to perform an analysis in which we consider genes that have either unstable or orphan repeats (or both) as repeat instable. For this analysis, we first identified 11,016 genes whose promoters are instable by this criterion in a tumor-normal pair. We found that MSI tumors have more genes with repeat instability than MSS tumors (2558 ± 274 and 2332 ± 364 genes, respectively, for promoter regions, WRS test, *P* = 0.15; 2371 ± 840 and 1109 ± 327, respectively, for exons, *P* = 0.003, see Fig. 3). We repeated this analysis also for repeats whose repeat unit consisted only of a single base (monucleotide repeats) to find that the difference in the number of genes with repeat instability between MSI and MSS genomes was now significant also for promoters (55 ± 4, 45 ± 11, respectively, for promoter region, *P* = 0.029 and 43 ± 28, 12 ± 7 respectively for exons, *P* = 10^−4^).

### Most cancer pathways in tumors are enriched for unstable and/or orphan repeats in gene promoters

**Fig. 4:**
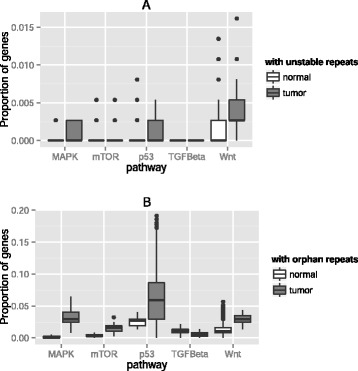
Gene promoters in most cancer pathways are significantly enriched for repeat instability. Box plots of the proportion of promoters in five different cancer-associated signaling pathways with **a**) unstable repeats in their promoters and **b**) orphan repeats in their promoters, for normal-normal genome pairs (left boxes, *n* = 595) and for tumor-matched normal genome pairs (right boxes, *n* = 35). The pathways are: MAPK (number of genes in the pathway, *N* = 113), mTOR (*N* = 44), p53 (*N* = 98), TGF beta (*N* = 29), and Wnt (*N* = 87). Thick horizontal lines in each box mark the median, edges of boxes correspond to the 25th and 75th percentiles, and whiskers cover 99.3 % of the data’s range. In panel **a** repeat instability is significantly different in tumor genomes for the MAPK, p53, and Wnt signaling pathways (WRS test, *P* = 0.03, *P* < 10^−8^, *P* < 10^−5^, respectively, after Bonferroni correction). In panel **b** repeat instability is significantly different in tumor genomes for the MAPK, mTOR, p53, and Wnt signaling pathways (WRS Test, *P* < 10^−31^, *P* < 10^−24^, *P* < 10^−9^, *P* < 10^−18^, respectively after Bonferroni, correction)

Many unique mutations in a cancer-associated signaling pathway have similar functional effects on the pathway [27, 28], which makes the analysis of entire pathways important to understand tumorigenesis. We focused on five well-studied signaling pathways that play a central role in carcinogenesis [13, 22]. These are the Wnt, TGF beta, MAPK, mTOR, and p53 pathways (see Methods and Additional file 1: Table S3). We identified 371 genes in these five pathways, and will refer to them for brevity as *cancer genes*. We first identified promoters of these genes with tandem repeats and asked how many of them contain orphan or unstable repeats. We found that in the MAPK, p53, and Wnt signaling pathways, there were significantly more genes with unstable repeats in their promoters (after Bonferroni correction [29], WRS test, *P* = 0.03, *P* < 10^−8^, *P* < 10^−5^, respectively, see Fig. 4a) between tumor-normal genomes compared to normal genome pairs. Similarly, in the MAPK, mTOR, p53, and Wnt signaling pathways, there were significantly more genes with orphan repeats (after Bonferroni correction, WRS test, *P* < 10^−31^, *P* < 10^−24^, *P* < 10^−9^, *P* < 10^−18^, respectively, see Fig. 4b) between tumor-normal genomes compared to normal genome pairs. Genes in the TGF beta pathway contained only stable repeats and very few orphan repeats both in the normal genome and the tumor-normal genome pairs. Nevertheless, these findings indicate that tumor genomes are overall enriched for unstable and/or orphan repeats in most cancer-associated signaling pathways.

**Fig. 5:**
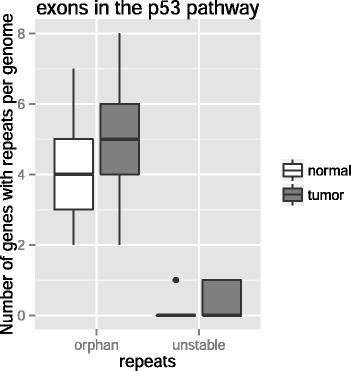
Exons in the p53 pathway are significantly enriched for repeat instability. Box plots of the number of genes in the p53 pathway with unstable repeats and orphan repeats in their exons, for normal-normal genome pairs (left boxes, *n* = 595) and for tumor-matched normal genome pairs (right boxes, *n* = 35). Thick horizontal lines in each box mark the median, edges of boxes correspond to the 25th and 75th percentiles, and whiskers cover 99.3 % of the data’s range. Repeat instability is significantly different between tumor and normal genomes (WRS test, *P* = 0.03, *P* < 10^−4^, respectively, after Bonferroni correction)

An analogous analysis on exons revealed that only the p53 pathway contains unstable or orphan exonic repeats, whereas genes in the other pathways do not show increased repeat instability. In addition, exons in the p53 pathway are significantly enriched for orphan and unstable repeats (after Bonferroni correction, WRS test, *P* < 10^−4^, *P* = 0.03, respectively, see Fig. 5). When we inspected the genes with repeats in this pathway more closely, we found three genes with both orphan and unstable genes in the tumor-normal genome pairs, which do not contain any such repeat in normal genome pairs. These genes are *TP53I3*, *TP53I11* and *CDKL1*.

### Genes with repeat instability are downregulated in tumors

**Fig. 6:**
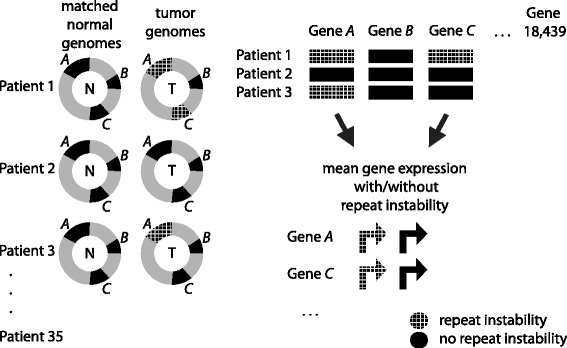
Schematic illustration of the expression analysis of genes with and without repeat instability. A gene (*A*, *B*, *C* and so on, where *n* = 18,439) is shown in solid black, if it does not contain any repeat instability (repeat copy number variation, repeat gain, or repeat loss) in its promoter or its exons between tumor and matched normal genomes of a patient (*n* = 35). It is shown cross-hatched if it contains at least one of these repeat instability types. Only genes with a repeat instability in at least one of the patients are considered for this expression analysis (*n* = 11,016 genes for promoter repeats, and *n* = 7531 genes for exonic repeats). For each gene, we computed (i) the mean of its expression level in tumors where the gene shows repeat instability (cross-hatched arrow indicating transcription) and (ii) the mean of its expression in tumors where the gene does not show repeat instability (solid black arrow indicating transcription). We then performed a WSR test to compare the mean expression values between these two groups of genes, the null hypothesis being that the expression level of a gene is not associated with mutations in its tandem repeat in a tumor tissue

**Fig. 7:**
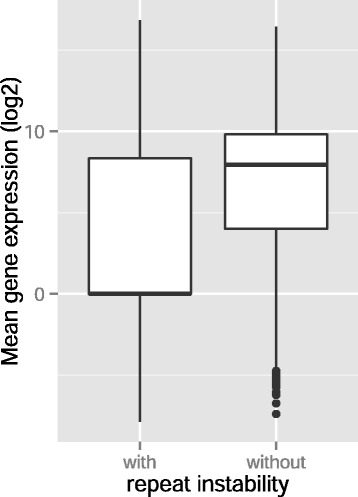
Genes with repeat instability are downregulated. Box plot of binary logarithm of mean expression levels of those genes with repeat instability (copy number variation, repeat gain, or repeat loss) (left box) and of genes without repeat instability (right box) in tumor genomes. Thick horizontal lines in each box mark the median, edges of boxes correspond to the 25th and 75th percentiles, and whiskers cover 99.3 % of the data’s range. The difference in gene expression is significant (WRS Test, *P* < 10^−350^, *n* = 11,016)

Next we asked whether repeat instability in tumors is associated with differential gene expression. More specifically, we wondered if an acquired somatic repeat mutation in a tumor can change a gene’s expression. Because the Cancer Genome Atlas (TCGA) Data Portal [13] contains RNA-seq based gene expression measurements mostly for tumor tissues, we compared a gene’s expression in a tissue where the gene acquired a repeat mutation (relative to the matched normal tissue) with the gene’s expression in a tissue where it did not acquire such a mutation. To this end, we analyzed the 11,016 genes whose promoters contain any of three possible instabilities (de novo repeat gain, repeat loss, or copy number variation) in at least one patient (see Fig. 6). Subsequently, we retrieved gene expression data for these genes in the 35 tumors [13]. For each gene, we then computed the binary logarithm of the mean expression level of the gene (i) in those genomes where the gene has a repeat instability compared to the normal tisssue and (ii) in genomes where the gene does not contain an unstable or orphan repeat. We then asked if there is a difference between the mean expression levels of these genes, with the null hypothesis that the expression level of a gene does not change when it acquires a somatic tandem repeat mutation in a tumor tissue (Wilcoxon signed rank (WSR) test [30]). We found that the mean expression levels differ significantly (*P* < 10^−350^): Genes whose promoters showed repeat instability were expressed at significantly lower levels (see Fig. 7). Results of an analogous expression analysis on exonic repeats also showed a slight but significant (WSR test, *P* = 0.0015) decrease in gene expression for exons showing a repeat instability.

### Pathway-specific gene expression alterations associated with repeat instability

**Fig. 8:**
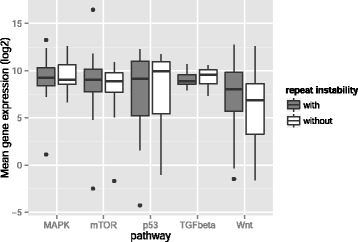
Genes with repeat instability are dysregulated in the Wnt, MAPK and p53 pathways. Box plot of binary logarithm of mean expression levels of those genes with repeat instability (copy number variation, repeat gain, or repeat loss) (left box in each pair) and of genes without repeat instability (right box) in tumor genomes, for genes in the Wnt (*n* = 32, for both boxes), TGF beta (*n* = 6), MAPK (*n* = 19), mTOR (*n* = 38) and p53 (*n* = 31) signaling pathways. Thick horizontal lines in each box mark the median, edges of boxes correspond to the 25th and 75th percentiles, and whiskers cover 99.7 % of the data’s range. Differences in gene expression are significant for the p53 (WSR test after Bonferroni correction *P* < 10^−4^), MAPK (*P* < 10^−5^) and Wnt signaling pathways (*P* = 0.03)

In the above analysis, we showed that genes with unstable repeats have significantly decreased expression levels. Because we wondered whether this also holds for each individual cancer pathway, we repeated our expression analysis for each pathway separately. To this end, we first identified a set of gene promoters with repeat instabilities for each pathway. Next, for each gene in a pathway-specific gene set, we computed the binary logarithm of the mean expression level of the gene in those genomes where the gene’s promoter shows repeat instability and compared it to the expression level of the same gene, but in genomes where the gene’s promoter does not show repeat instability. Comparing these average gene expression levels, we found that genes with tandem repeat instabilities in their promoters are significantly downregulated in the p53 pathway (WSR test after Bonferroni correction for multiple testing, *P* < 10^−4^) and overexpressed in the MAPK and Wnt signaling pathways (*P* < 10^−5^, *P* = 0.03, respectively). In contrast, genes in the TGF beta (*P* = 0.56) and the mTOR (*P* = 0.77) pathways did not show any significant difference (see Fig. 8). We repeated this analysis with exons in the p53 pathway, because it is the only cancer pathway that contained unstable or orphan repeats in the exonic sequences. We observed slightly decreased levels of gene expression, when a gene in this pathway contains unstable or orphan repeats, but the difference was not significant (*P* = 0.25).

## Discussion

Here we present a comprehensive analysis of exome and whole genome sequencing data from 35 patients with colorectal cancers to identify tandem repeat instabilities and their association with gene expression alterations. To our knowledge, this is the first genome-wide analysis of tandem repeat instabilities in the currently largest collection of colorectal tumor genomes. To date, there are few other studies on genome-wide tandem repeat mutations in cancers. One such study [31] genotyped repeat variations in breast cancer exomes in comparison to random healthy individual tissues. Another study [7] focuses on microsatellite mutations within various tumors (including colorectal cancer) in a small number of genes (137). Our study remains unique in its focus on comparison between tissues from the same individual.

Using the matched tumor and normal tissues, we identified two types of repeat instability between these pairs of genomes, namely (i) repeat copy number variation, and (ii) de novo gains and losses of repeats. We identified these instabilities both in promoter regions and exons of 18,439 human genes, and in a smaller set of 371 genes from five signaling pathways associated with cancer. We found evidence for enhanced repeat instability in promoters and exons of tumor tissues. We also showed for the first time that tumor genomes with an MSI phenotype, which indicates a defect mismatch repair system, contain more repeat instabilities than microsatellite stable tumors. The difference was more pronounced when we focused on mononucleotide repeats, in agreement with the finding that replication slippage alone cannot explain the incidence of polymorphisms in repeats whose repeat units are longer than one nucleotide [32]. Although replication slippage is a major factor driving mononucleotide repeat variation, additional cellular factors, such as chromatin reorganization [33] and telomere instability [34] also play a role for non-mononucleotide repeats.

Motivated by the impact of gene regulatory alterations on carcinogenesis, we studied repeat-associated gene expression changes. Using the comprehensive catalogue of information we retrieved from [13], we compared a gene’s expression level in genomes where the gene shows repeat instability and where it does not. We observed that genes with repeat instability are mostly downregulated, and especially so if this instability occurred in the promoter, emphasizing the importance of regulatory mutations in carcinogenesis also suggested by others. Two other studies [15, 35] identified recurrent mutations in gene promoters and their association with gene expression levels in multiple tumor genomes across many cancer types. Another study on non-coding disease associated variants [36] showed that these variants are concentrated in regulatory DNA marked by DNase hypersensitive sites and that these variants perturb epigenetic processes.

Gene silencing mediated by repeats is a phenomenon observed in various diseases, where, for example, DNA around tandem repeats becomes heterochromatic, leading to decreased promoter accessibility and hence to local transcription repression. This phenomenon has been documented in mammalian embryonic carcinoma cells [37], as well as for repeat-induced diseases such as myotonic dystrophy and Friedreich’s ataxia [38]. Apart from chromatin reorganization, promoter hypermethylation, which is commonly observed in carcinogenesis [18, 39, 40], can also cause gene silencing or reduced gene expression. Several genes are downregulated via promoter hypermethylation in colon cancers [39, 41]. This type of downregulation can act synergistically with other genetic mechanisms, such as somatic mutations, to alter key signaling pathways critical to colorectal tumorigenesis [39, 42]. Previous smaller-scale studies based on the five markers characterized in the “Bethesda guidelines” microsatellites [23] showed an association between promoter hypermethylation and microsatellite instability [43, 44]. We therefore asked if promoters with repeat instability show higher promoter methylation levels, and found indeed a small but significant increase of methylation in promoters with unstable repeats (WSR test, *P* = 0.004, see Methods and Additional file 2: Figure S1). Our findings reveal, for the first time, a genome-wide association between promoter methylation and decreased expression in genes with repeat instability.

Although identification of mutated cancer genes provides insights into tumorigenesis [45], diverse and functionally heterogeneous genes can be mutated even within same type of tumor [27, 28]. However, some pathway dysregulations are shared among multiple cancer types [22, 27, 46]. We therefore identified unstable repeats in the promoters of genes in five prominent cancer-associated pathways. One of them is the Wnt signaling pathway, which is commonly implicated in carcinogenesis due to its regulatory role in cell proliferation, gene transcription and cell migration [13, 47]. Colorectal cancers of all subtypes almost invariably start with an activating mutation in this pathway [22, 48]. Remarkably, we found that gene promoters in the Wnt pathway are significantly enriched for unstable and/or orphan repeats, and these genes are also significantly overexpressed (in contrast to the opposite genome-wide trend discussed above). Genes in the MAPK pathway, a signaling cascade that regulates cellular transcription and translation levels [27], also show higher repeat instability in the promoters of tumor-normal genome pairs than of normal genome pairs, and those promoters with unstable and/or orphan repeats are also significantly overexpressed. This increase in gene expression is in line with the significant hyperactivation of the MAPK pathway revealed by a comprehensive study on colorectal tumors by TCGA [13]. In contrast, none of the genes in the TGF beta pathway show increased repeat instability or expression alterations that are associated with repeat instability. This observation is in line with the previous observation [13] that this pathway is the least divergent pathway between colorectal tumors and their matched normal genomes in terms of gene copy number variation and gene expression.

The final pathway we analyzed is the p53 pathway. It plays a crucial role in the cell cycle and can initiate cell death [49]. Inactivation of the p53 pathway through multiple mutations is an almost universal feature of human cancer cells [50, 51]. In agreement with its central role in tumor suppression, we found that genes in this pathway are significantly enriched both for promoter and exonic repeat instabilities in tumor-normal pairs compared to normal genome pairs, and genes with instabilities both in promoter and exon sequences are downregulated in colorectal tumors. When we examined pathway genes with exonic repeats, we identified several genes with unstable and/or orphan repeats in tumor-matched normal genome pairs but not in normal genome pairs. One of them, *TP53I3* is a well-known example for tandem repeat instability associated with cancer. It has been shown that this gene contains a pentanucleotide (TGCCC) repeat where the tumor-suppressor *p53* binds to activate the gene, a mechanism suggested to be mediating cell death [52]. Copy number variation in this repeat alters *TP53I3* activation and probably affects an individual’s susceptibility to cancer [53]. We show for the first time that this repeat is actually polymorphic in a tumor tissue. We also identified two other genes (*TP53I11* and *CDKL1*) that contain tumor-specific repeat instabilities in their exons, and where repeat instability had not been documented so far. These findings highlight the importance of analyzing tumor-specific tandem repeat instability, and their consequences on gene regulation, which could contribute to carcinogenesis.

Among the limitations of our study is that we cannot distinguish between somatic and germline mutations. This is relevant, because some mismatch repair genes can experience germline mutations that cause colorectal cancer [10]. These germline mutations can also play a role in forming different subtypes of colorectal cancer, as they trigger accumulation of different sets of somatic mutations throughout carcinogenesis [22]. However, because 90 % of cancer mutations are somatic [45], this is not a serious drawback. Second, an ideal control analysis would compare repeat instability between normal-normal genome pairs from healthy tissues of the *same* individual to those of tumor-normal genome pairs. However, the necessary multiple normal genomes are currently not available, which is why we had to compare the genomes of normal tissues from *different* individuals as a control. As a result, we may underestimate differences in repeat instability between normal and tumor genomes. Another source of underestimating repeat number and instability is our conservative approach of identifying matched repeats (see Methods). Absent these limitations, we might see an even greater excess of unstable repeats in colorectal tumors. Some of them would be by-products of defective mismatch repair, whereas others might trigger or promote carcinogenesis. It is also important to note that our cancer gene set is unlikely to encompass all genes that may play a role in cancer, because we focused on particular, well studied cancer associated pathways. Finally, limitations in whole genome alignment quality may underestimate repeat copy number variation in gene promoters.

## Conclusions

Because genetic instability is not only central to tumor pathogenesis, but may also underlie the development of resistance to chemotherapeutic agents, it is important to identify its incidence and phenotypic consequences. Our analysis, based on the best currently available data sets is a first small step towards this understanding. Future studies using more data and more advanced technologies will enhance this understanding further, in order to develop more effective molecular diagnostic approaches centered on repeat instability. For example, studies comparing gene expression levels between tumor and healthy tissues will be able to identify tumor-specific gene expression alterations more confidently. Also, information on allele-specific expression can help explaining the association between repeat instability and downregulation. Future studies with a more comprehensive set of microsatellite stable tumors will hopefully disentangle differences between microsatellite-stable and -unstable tumors in greater detail. Finally, differentiating between clonal and subclonal instabilities will facilitate a better understanding of the life histories of tumors, because they show extreme intra-tumor heterogeneity [54–56].

## Methods

### Genome sequence analysis

We obtained whole genome sequences of colon and rectal tumors, together with their matched genomes -- the same individual’s genomic sequences from blood samples -- from the controlled access data tier of the Cancer Genome Atlas Data Portal (TCGA, http://cancergenome.nih.gov/, [13]). For our analysis, we considered only genomes for which RNA-Seq data were also available in TCGA. The genome sequence data is based on 2-5X coverage Illumina HiSeq2000 sequencing of 80–100 million pairs of 100-nucleotide-long reads, aligned against human genome build #18 [13] with the indel-compatible software package BWA (bwa-0.5.9rcl [57]). For the exon analysis, we used Illumina exome-seq data exceeding 20X coverage for ~44Mbs of sequence from ~30 K genes.

We generated consensus sequences for the promoters and exons of genes in the tumors and their matched normal genomes using SAMtools [58]. In order to specify the exonic regions, we considered all transcript variants for each gene in the human reference genome annotation [59] for human genome build #18. We excluded those exons that contained transcript variants in more than one chromosome, such as transposons. For genes with multiple transcripts, we merged all exonic regions from all transcripts into one super-transcript. Because our previous work on human tandem repeats [60] suggests that the 5,000 base pairs [bps] upstream from the transcription start site contain the most regulatory signals, we focused on this region and refered to it as the promoter.

While generating our consensus sequences, we noticed that some genomes contained many more unaligned sequences than others. We eliminated genomes with unaligned nucleotides in more than 10 % of the regions of our interest (promoters or exons), which reduced our data set to 35 genomes (see Additional file 1: Table S2 for a list of genomes). We considered a tumor MSI, if its MSI status was MSI-H based on [13]. Because this approach yielded only three genomes, we considered also other criteria of an MSI phenotype, as provided by [13]. We found, however, only one more genome that was not MSI-H but showed all other indications of an MSI phenotype, namely a CIMP-H methylation subtype, MLH1 silencing, and a MSI-CIMP expression subtype. We therefore considered this genome also MSI (see Additional file 1: Table S2). After removing genes from the data set whose promoters or exons could not be aligned, we focused our analysis on the remaining “global” set of (one-to-one homologous) 18,439 genes (see Additional file 1: Table S1), as listed in [13]. Apart from analyzing this global set, we also performed a more detailed analysis of 371 cancer genes (Additional file 1: Table S3) that fall into five well-studied cancer associated pathways [13, 22, 28].

### Tandem repeat identification

We used the program Tandem Repeat Finder 4.07b [61] to identify tandem repeats in the consensus sequences. Specifically, we identified repeats with (i) an incidence of indels (insertions or deletions) in adjacent repeat units below 10 % (e.g., a repeat unit of 20 nucleotides can have up to two single base pair indels relative to the consensus pattern, which is the repeat unit most common in the whole repeat sequence [61]), and (ii) a sequence identity of repeat units above 90 % (e.g., at least 18 nucleotides of a repeat unit of 20 nucleotides must match the consensus pattern). We set the Tandem Repeat Finder Score to a value of 80, as we were most interested in how repeat variation might cause gene expression differences, and variation of tandem repeats increases strongly for repeats of high Tandem Repeat Finder Scores [62]. We considered both micro- and minisatellites with tandem repeat units up to 100 nucleotides in length. Repeats longer than that are more stable and therefore less likely to cause gene expression differences [62].

To identify repeat gains and losses, we first defined matched repeats between a tumor and its normal genome. These are repeats with the same repeat unit that occur in the promoter or exon of the same (homologous) genes in a tumor and its matched normal genome. We did not consider gene families separately. We allowed positional variation of repeats up to 50 nucleotides within a promoter or an exon, because indels can cause substantial shifts in repeat location even within a species [63].

To find out whether a tumor genome shows a significant difference in repeat incidence or variability to a normal genome, it is necessary to compare (i) the incidence or variability of repeats in a tumor genome relative to its matched normal genome to (ii) the incidence or variability of repeats between two normal genomes. We computed the latter from our 35 normal genomes by pairing them in all possible (595) combinations, computing our measures of repeat incidence and variability for each pair, and pooling the resulting data.

### Gene expression analysis

The gene expression data we used is based on RNA sequencing of 350–450 base pair-long Illumina Cluster Station and Genome Analyzer reads by TCGA [13]. The data comprises expression levels in reads per kilobase of transcript per million reads mapped (rpkm) for 18,439 genes in the 35 tumor genomes we analyzed.

### Methylation analysis

The promoter methylation data we used is based on Illumina Infinium HumanMethylation27 arrays to profile DNA methylation at gene promoters of TCGA [13], targeting 27,578 CpG sites located in proximity to the transcription start sites of 14,475 consensus coding sequencing (CCDS) in the NCBI Database (Genome Build 36). We computed for each gene the methylation level in those genomes where the gene has a repeat instability in its promoter, and compared it to the gene’s methylation level in genomes where the gene has no repeat instability.

## Additional files

Additional file 1:
**This file contains 3 supplemental tables.** (XLSX 263 kb) Additional file 2:
**This file contains 1 supplemental figure.** (PDF 39 kb)
