# Supplementary material for: A survey of tandem repeat instabilities and associated gene expression changes in 35 colorectal cancers
Source: BMC Genomics. 2015 Sep 16;16(1):702. doi: 10.1186/s12864-015-1902-9 (PMC4574073; doi:10.1186/s12864-015-1902-9)
Supplement: Additional file 2: — This file contains 1 supplemental figure. (PDF 39 kb) [file 12864_2015_1902_MOESM2_ESM.pdf]

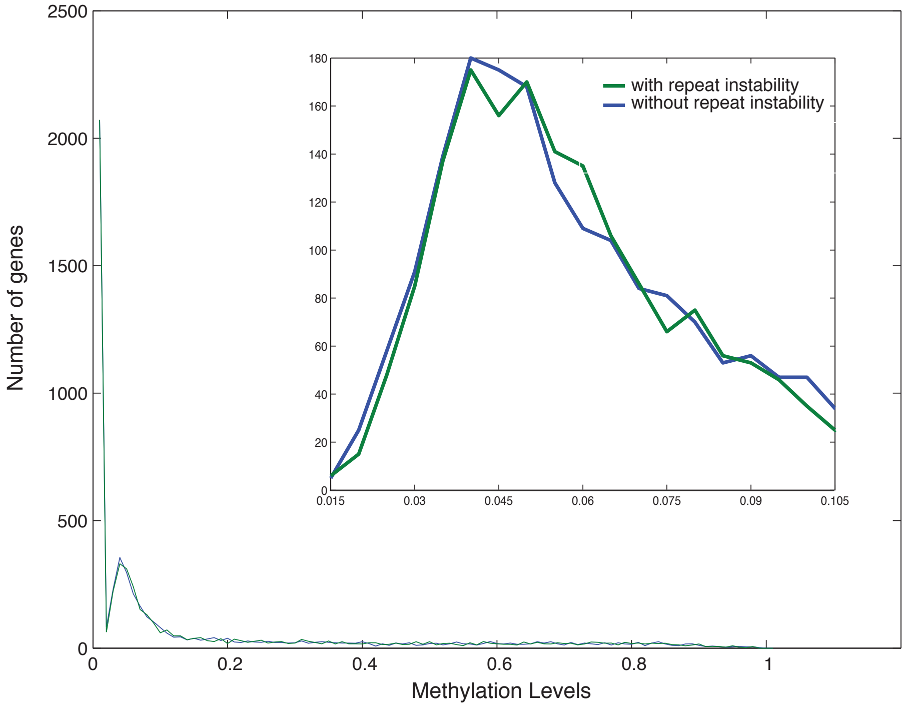

Supplemental Figure 1. Increased methylation in genes with repeat instability. Histogram of promoter methylation levels for genes with repeat instability (green line) and for genes without repeat instability (blue line). The inset focuses on a narrow window of methylation levels (0.015-0.1), where most of the data points are found.
